# BLNK mutation associated with T-cell LGL leukemia and autoimmune diseases: Case report in hematology

**DOI:** 10.3389/fmed.2022.997161

**Published:** 2022-11-16

**Authors:** Guillemette Fouquet, Julien Rossignol, Laure Ricard, Flavia Guillem, Lucile Couronné, Vahid Asnafi, Manon Vavasseur, Mélanie Parisot, Nicolas Garcelon, Frédéric Rieux-Laucat, Arsène Mekinian, Olivier Hermine

**Affiliations:** ^1^Hématologie Clinique, Centre Hospitalier Sud Francilien, Corbeil-Essonnes, France; ^2^Imagine Institute, INSERM U1163, Université de Paris, Paris, France; ^3^Institut Cochin, INSERM, Centre National de la Recherche Scientifique (CNRS), Université de Paris, Paris, France; ^4^Department of Adult Hematology, Hôpital Universitaire Necker-Enfants Malades, Assistance Publique des Hôpitaux de Paris, Paris, France; ^5^Department of Internal Medicine and Inflammatory Diseases, Hôpital Saint Antoine, Assistance Publique des Hôpitaux de Paris, Sorbonne University, Paris, France; ^6^Laboratory of Excellence GR-ex, Paris, France; ^7^Institut Necker-Enfants Malades (INEM), INSERM U1151, Université de Paris, and Laboratory of Onco-Hematology, Hôpital Universitaire Necker-Enfants Malades, Assistance Publique des Hôpitaux de Paris, Paris, France; ^8^Genomics Core Facility, Institut Imagine-Structure Fédérative de Recherche Necker, INSERM U1163, Paris, France; ^9^Immunopathology/Biotherapy Department (DHU i2B), UMR 7211, Sorbonne University, Paris, France

**Keywords:** B-cell linker, large granular lymphocyte leukemia, autoimmune diseases, regulatory B-cells, regulatory T-cells (T reg), case report

## Abstract

We present the case of a female patient with a heterozygous somatic BLNK mutation, a T-cell LGL (large granular lymphocyte) leukemia, and multiple autoimmune diseases. Although this mutation seems uncommon especially in this kind of clinical observation, it could represent a new mechanism for autoimmune diseases associated with LGL leukemia. The patient developed several autoimmune diseases: pure red blood cell apalsia, thyroiditis, oophoritis, and alopecia areata. She also presented a T-cell LGL leukemia which required treatment with corticosteroids and cyclophosphamide, with good efficacy. Interestingly, she had no notable infectious history. The erythroblastopenia also resolved, the alopecia evolves by flare-ups, and the patient is still under hormonal supplementation for thyroiditis and oophoritis. We wanted to try to understand the unusual clinical picture presented by this patient. We therefore performed whole-genome sequencing, identifying a heterozygous somatic BLNK mutation. Her total gamma globulin level was slightly decreased. Regarding the lymphocyte subpopulations, she presented a B-cell deficiency with increased autoreactive B-cells and a CD4+ and Treg deficiency. This B-cell deficiency persisted after complete remission of erythroblastopenia and LGL leukemia. We propose that the persistent B-cell deficiency linked to the BLNK mutation can explain her clinical phenotype.

## Introduction

Large granular lymphocyte leukemia is a rare chronic mature lymphoproliferative disorder of either T-cell or NK lineage characterized by a clonal expansion of LGLs-resistant to activation-induced cell death. The exact initiation process of LGL leukemia is still unknown; however, the involvement of autoimmune and inflammatory processes in the pathology is generally accepted. LGL leukemia can indeed be associated with various autoimmune diseases—primarily rheumatoid arthritis, autoimmune thyroiditis, or lupus erythematosus (1). In these cases, LGL leukemia can be seen as a consequence of the autoimmune disease or as a secondary lymphoproliferative disease.

*STAT3* mutations are the most common mutations to date in LGL leukemia and can be found in 30–40% of cases (2). More recently, many mutations have been described in this rare disease, especially affecting the JAK/STAT pathway such as mutations in STAT5b (3–6). However, LGL leukemia remains a clinically and genetically heterogeneous disease, and genomic analysis could allow a better understanding of the molecular mechanisms of the LGL leukemia and of the associated autoimmune diseases, and the identification of new treatments.

## Patient information

The patient was born on 1974. She was diagnosed with ovarian failure with early menopause revealing autoimmune oophoritis in 1990 (16 years old), for which she receives hormone replacement. She presented alopecia areata in the following years, treated by topical corticosteroids. She was then treated in hematology as described below, for autoimmune pure red cell aplasia and LGL leukemia in 2011 (37 years old). In 2015, she developed hypothyroidism linked to Hashimoto's thyroiditis (41 years old), treated by hormone replacement.

She had no relevant infectious history. She has no other significant medical nor psychosocial history.

There is no particular pathology in her family, nor consanguinity.

## Clinical findings

The patient first consulted in hematology at 37 years old. She presented with anemia (hemoglobin 8.3 g/dL) and neutropenia (neutrophils 0.7 G/L).

The blood smear showed circulating large granular lymphocytes, and immunophenotyping and molecular biology confirmed T-cell LGL leukemia. Bone marrow analysis revealed pure red blood cell aplasia (PRCA).

## Timeline

**Figure 1:**
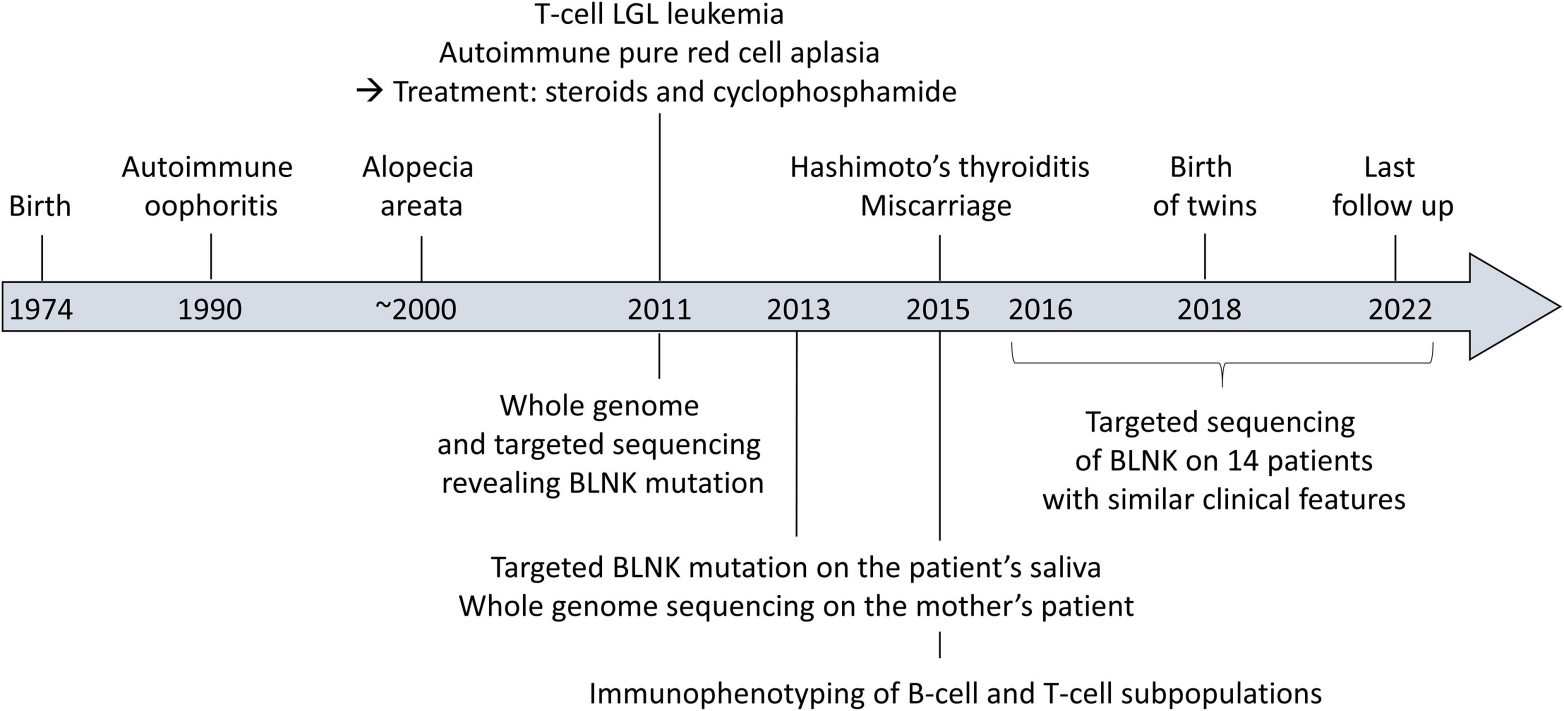
Timeline of the clinical case.

Timeline of the clinical case is presented Figure 1.

## Diagnostic assessment

### Immunophenotyping and protein electrophoresis

The blood count revealed a total lymphocyte count of 1,400/mm^3^. The blood smear showed large granular T-cells. At diagnosis, immunophenotyping confirmed an excess of large granular T-cells (700/mm^3^), along with B-cell lymphopenia (CD19^+^ cells: 24/mm^3^; normal range: 169–271), and a moderate decrease in CD4^+^ T-cells (CD4^+^ cells: 387/mm^3^; normal range: 460–1,232). The LGL T-cells were CD2+, CD3+, CD5low, CD7low, CD4-, CD8+, CD57+, CD16+, CD56-, CD62L-. Clonality assessment found a restricted expression of Vb3.1, and molecular biology confirmed monoclonality.

Her total gamma globulin level was 6.9 g/L (normal range: 8–13.5), IgG 4.8 g/L (normal range: 6.65–12.78), IgA 1.6 g/L (normal range: 0.7–3.45), and IgM 0.4 g/L (normal range: 0.5–2.09).

### Immunophenotyping of B-cell and T-cell subpopulations

Of note, these B- and T-cell analyses were performed while the patient was in complete remission of PRCA and LGL leukemia, without any immunosuppressive therapy.

**Figure 2:**
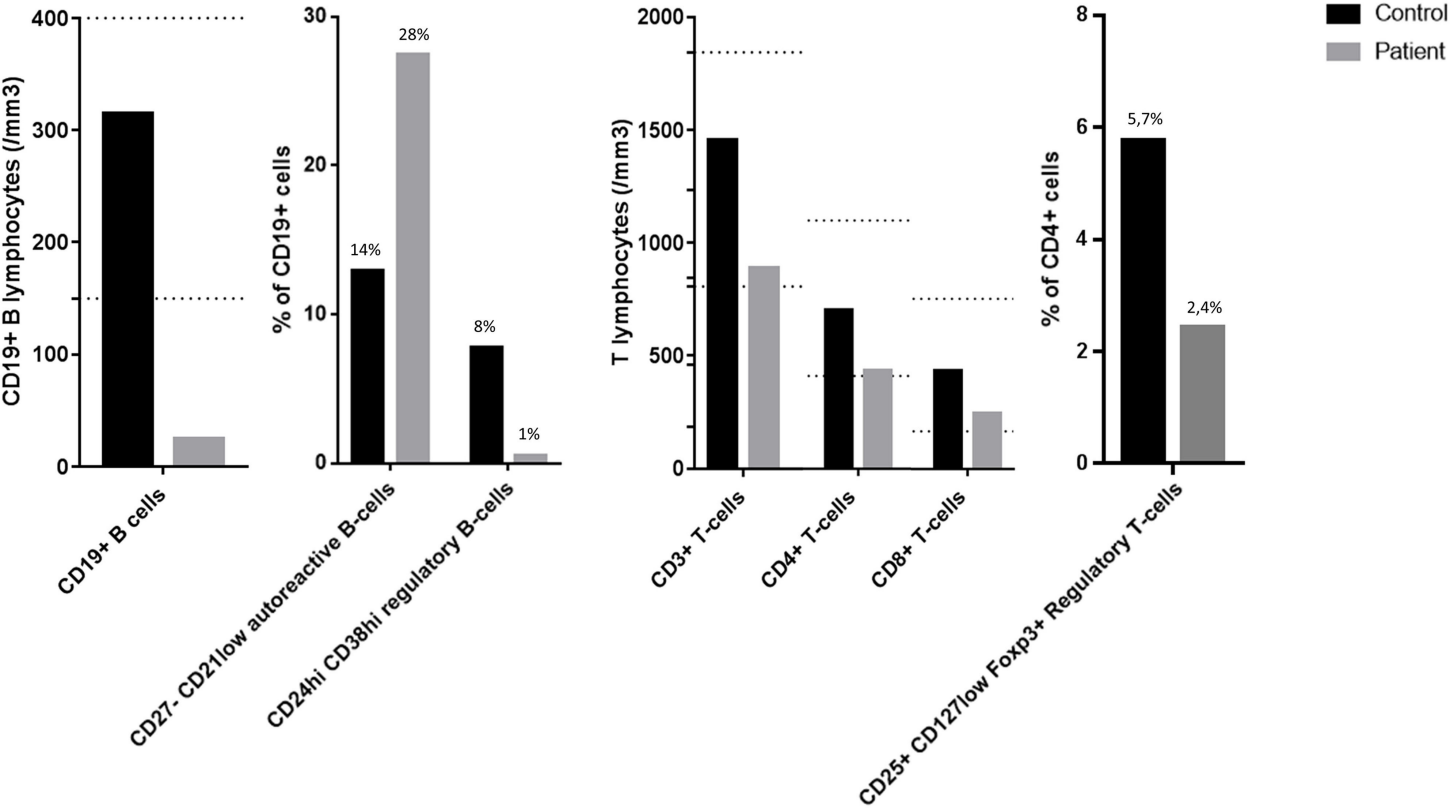
Flow cytometry analyses of B-cell and T-cell subpopulations. The dotted lines represent the normal range of the laboratory: CD19+ B lymphocytes 169–271/mm^3^; CD3+ T lymphocytes 807–1844/mm^3^; CD4^+^ T lymphocytes 460–1232/mm^3^; CD8^+^ T lymphocytes 187–844/mm^3^.

Analysis of B-cell and T-cell subpopulations revealed persistent and profound B-cell lymphopenia with conserved distribution of various B-cell subpopulations (memory, transitional, and naive B-cells), along with an increased proportion of CD27^−^CD21^low^ B-cells, previously described as autoreactive B-cells (7). Breg analyses showed a reduction in CD19^+^CD24^hi^CD38^hi^ cells but no significant drop in other Breg populations (i.e., IL10 producing B-cells, CD24^hi^CD27^+^ (B10) and CD27^int^CD38^hi^ plasmablasts) (8). T-cell population analyses only showed a moderate CD4^+^ and Treg deficiency (Figure 2).

Unfortunately, the patient's B-cell lymphopenia made functional exploration of the B-cell and T-cell populations inconclusive.

Flow cytometry gating strategies are presented in Supplementary Figure 1.

### Whole genome sequencing

This unusual association of multiple autoimmune diseases prompted us to perform whole-exome sequencing (WES) for this patient in order to search for any predisposing mutation. WES was performed on a blood sample, while the patient still presented circulating T LGL clonal cells. Analysis revealed the presence of a heterozygous nonsense mutation in the BLNK gene (c.1,102 C>T, p.Q368^*^, 47%) that yielded a premature STOP codon. The mutation was located in the BLNK C-terminal SH2 domain, which allows recruitment of BLNK to the BCR complex through its interaction with BTK.

No STAT3 mutation was found, neither by WES nor using targeted sequencing. No mutation was found in the JAK/STAT pathway by WES.

To understand the development of LGL leukemia in this patient, we performed comparative exome analysis of sorted T-cells (CD3^+^) and B-cells (CD19^+^). However, no mutation other than BLNK that could explain a proliferative advantage was found in T-cells. This could suggest that the T-LGL proliferation, even clonal, was reactive in this autoimmune context.

WES was also performed on a blood sample of the patient's mother: no BLNK mutation was found.

### BLNK-targeted sequencing

Targeted BLNK sequencing on sorted medullary populations—T-cells (CD3^+^), B-cells (CD19^+^), monocytes (CD14^+^), and others (CD3^−^ CD19^−^ CD14^−^)—revealed the same heterozygous BLNK mutation in all cell groups.

The mutation was not found by targeted BLNK sequencing on the patient's saliva, demonstrating that it is a somatic and not a germline mutation. These findings are in accordance with a somatic mosaicism, the BLNK mutation presumably having occurred during the embryonic, fetal, or postnatal period. Such a mechanism has already been described in the context of lymphoproliferative autoimmune syndromes (9).

**Figure 3:**
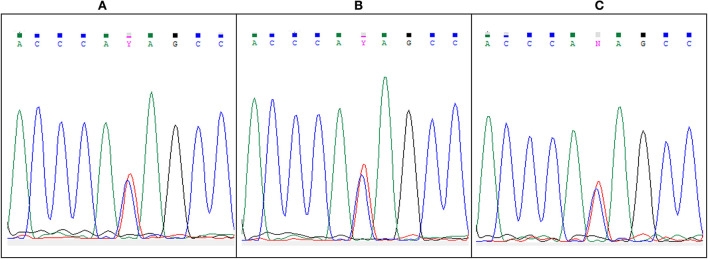
Heterozygous C > T BLNK mutation revealed by Sanger sequencing of BLNK in **(A)** whole blood at diagnosis, and in **(B)** T-cells and **(C)** non–T-cell PBMCs at remission.

In line with this hypothesis, the heterozygous BLNK mutation was still present in sorted peripheral blood mononuclear cells—T-cells (CD3+) and others (CD3-)—after complete remission of the LGL leukemia (Figure 3).

### BLNK-targeted sequencing in patients with similar clinical manifestations

In order to investigate whether this acquired BLNK mutation could be present in other patients, we performed targeted sequencing of BLNK on 14 patients presenting similar clinical features: clonal LGL and autoimmune disease(s). Characteristics of patients are presented in Table 1. DNA samples were available from the Hôpital Universitaire Necker-Enfants Malades LGL leukemia cohort and a computerized database (Dr Warehouse; N. Garcelon) (10). No BLNK mutation was found in this 14 patients cohort.

**Table 1:** Characteristics of patients with BLNK-targeted sequencing.

	**Age at LGL diagnosis**	**Sex**	**Type of LGL**	**Treatment of LGL**	**Associated autoimmune diseases**	**Treatment of autoimmune diseases**	**Source of DNA**
1	38	F	T LGL	Methotrexate	Rheumatoid arthritis	Adalimumab, Rituximab, Abatacept, Tocilizumab	Blood
2	41	M	T LGL	No added treatment	Immune thrombocytopenia	Steroids, Immunoglobulins, Splenectomy, Rituximab Vincristine, Cyclophosphamide, Romiplostim	Bone marrow
3	54	F	T LGL	Cyclosporine Methotrexate	Autoimmune enteropathy	Local (intestinal) steroids	Blood
4	73	M	T LGL	Methotrexate	Rheumatoid arthritis, Immune thrombocytopenia	Rituximab, Leflunomide	Blood
5	87	F	T LGL	Methotrexate	Cold agglutinin disease	Steroids, Plasma exchange, Cyclophosphamide, Chloraminophene, Interferon, Vesanoid, Fludarabine, Rituximab	Blood
6	70	F	T LGL	Methotrexate Cyclosporine Cyclophosphamide	Vasculitis, Autoimmune hemolytic anemia	Colchicine, Hydroxychloroquine	Blood
7	58	F	T LGL	Cyclosporine	Rheumatoid arthritis	Salazopyrin, Methotrexate, Hydroxychloroquine, Steroids, Leflunomide	Bone marrow
8	33	M	NK LGL	Cyclophosphamide	Autoimmune hemolytic anemia	Steroids	Blood
9	61	F	T LGL	Methotrexate	Rheumatoid arthritis, Sjögren's syndrome, Pyoderma gangenosum	Steroids, Methotrexate	Blood
10	50	M	T LGL	No added treatment	Autoimmune hemolytic anemia	Steroids, Rituximab, Splenectomy	Blood
11	72	F	T LGL	No added treatment	Celiac disease	Steroids	Blood
12	79	M	T LGL	No added treatment	Rheumatoid arthritis	Steroids, Hydroxychloroquine	Blood
13	36	M	T LGL	Steroids	Autoimmune hemolytic anemia, Immune thrombocytopenia	Steroids, Hydroxychloroquine, Immunoglobulins, Rituximab, Splenectomy	Blood
14	58	F	T LGL	Methotrexate	Inflammatory rheumatism	Steroids, Methotrexate	Blood

To note, no BLNK mutation was found in a PRCA cohort at Imagine Institute (data not shown).

## Therapeutic intervention

The patient was treated for PRCA and LGL leukemia: she received corticosteroids at a dose of 1 mg/kg/day for 4 weeks for PRCA, before gradual decrease over 4 additional weeks. As the LGL leukemia was associated with neutropenia and an auto-immune disease, the patient also received, at the same time, cyclophosphamide (100 mg/day orally) over 6 months.

This treatment resulted in a rapid complete remission of PRCA and concomitant disappearance of the LGL clone in < 6 months.

## Follow-up and outcomes

The patient is still in complete remission of PRCA and LGL leukemia.

Her alopecia improved after dermatologic treatment including topical corticosteroids, but evolves by flare-ups.

She is still receiving hormone replacement for her thyroiditis and oophoritis. She had a miscarriage but then gave birth to twins after receiving an oocyte donation. She and her children were doing well at the last follow-up.

## Discussion

BLNK is an adaptor protein expressed in B-cells and macrophages that plays a major role in BCR signaling. Mutations affecting the pre-BCR signaling pathway result in severe B-cell differentiation blockades at the pre-B1 to pre-B2 cell transition, leading to primary B-cell immunodeficiencies characterized by the total or near-total absence of circulating B-cells, severe hypo- or agammaglobulinemia, and recurrent bacterial infections (11). BLNK KO mice display stymied pre-B-cell development and reduced numbers of mature B-cells, in addition to autoimmune diseases associated with lower B-cell and Treg percentages (12).

In humans, the function of BLNK in B-cells seems non-redundant (13). A few cases of homozygous BLNK mutations have been described, all leading to near-total absence of circulating B-cells and severe hypo- or agammaglobulinemia (11, 13–16). A dysfunction of BLNK has also been associated with autoimmune diseases, such as multiple sclerosis (17, 18), or rheumatoid arthritis which is known to be associated with LGL leukemia (19).

As far as we know, this is the first report about a symptomatic heterozygous BLNK mutation. Our patient presented an association of multiple autoimmune disorders, whose pathogenic mechanism is not fully understood but seems to involve B-cells—as is usually the case in other idiopathic autoimmune conditions and BLNK KO mice.

Her mutation leads to a premature STOP codon and is located in the BLNK C-terminal SH2 domain, which allows recruitment of BLNK to the BCR complex through its interaction with BTK. These data imply that the mutation necessarily impacts BLNK function. Unfortunately, the patient presented with a profound B-cell lymphopenia which made our functional assays inconclusive, but is consistent with a BLNK defect leading to an altered B lymphopoiesis. In proportion, all populations were preserved including Bregs, but there was a relative increase in autoreactive B-cells. As described elsewhere, an alteration of BLNK could lead to a dysregulation of B-cells with activation and selection of autoreactive B clones responsible for autoimmune manifestations (20). As her mutation appears to be somatic but is not expected to give a proliferative advantage, we believe that it may have occurred early in development. Since the BLNK alteration involves the pre-B stage, there could be a negative selection defect either centrally, leading to an enrichment of autoreactive B-cells, or peripherally with abnormal B-cells surviving in the periphery by escaping the GC cycle. However, further work is needed to resolve this very interesting issue.

She also presented with T-LGL, which might be reactive to the autoimmune diseases and chronic excess of cytokines production (such as IL-15) since BLNK is not expressed in T-cells. Alternatively, we cannot rule out that invalidated BLNK B-cells may interact with T-cells and induce their proliferation. In agreement with this hypothesis, it has recently been demonstrated that the anti-CD20 monoclonal antibody rituximab, a specific anti–B-cell drug, is somewhat effective in T-LGL associated with rheumatoid arthritis (21).

In conclusion, here we have presented the case of a female patient with a heterozygous somatic BLNK mutation, T-LGL, and multiple autoimmune diseases, and we propose that persistent B-cell deficiency linked to the BLNK mutation primarily explains her clinical phenotype.

## Patient perspective and informed consent

The treatment for PRCA and LGL leukemia was well tolerated. The patient is doing well today. The most painful problem for her was her ovarian failure, but she was very happy to give birth to two children thanks to an oocyte donation.

She gave informed consent for all analyses performed and for this manuscript, and repeatedly mentioned her enthusiasm for helping research.

## Data availability statement

The datasets presented in this article are not readily available because of ethical and privacy restrictions. Requests to access the datasets should be directed to the corresponding author.

## Ethics statement

Ethical review and approval was not required for the study on human participants in accordance with the local legislation and institutional requirements. The patients/participants provided their written informed consent to participate in this study. Written informed consent was obtained from the individual(s) for the publication of any potentially identifiable images or data included in this article.

## Author contributions

JR and OH cared for the patient. GF, JR, LR, FG, LC, AM, FR-L, NG, and OH conceived and planned the experiments. GF, LR, FG, MV, and MP performed the experiments. NG provided the computerized database used to select the 14 patients cohort. VA provided the samples for the 14 patients cohort. GF, JR, AM, and OH contributed to analysis, interpretation of the results, and wrote the original manuscript. GF, FR-L, and OH revised the manuscript. All authors contributed to the article and approved the submitted version.

## Funding

GF was supported by the Institut National du Cancer (INCA); and JR, through a PhD fellowship from the French Ministry of Higher Education and Research. Funding for whole genome sequencing came from the GR-Ex Laboratory of Excellence (ANR-11-LABX-0051) and the Imagine Institute. The Imagine Institute and Labex GR-Ex are funded by the Investissements d'Avenir program of the French National Research Agency (ANR-10-IAHU-01 and ANR-11-IDEX-0005-02, respectively). Publication fees were provided by Imagine Institute.

## Conflict of interest

The authors declare that the research was conducted in the absence of any commercial or financial relationships that could be construed as a potential conflict of interest.

## Publisher's note

All claims expressed in this article are solely those of the authors and do not necessarily represent those of their affiliated organizations, or those of the publisher, the editors and the reviewers. Any product that may be evaluated in this article, or claim that may be made by its manufacturer, is not guaranteed or endorsed by the publisher.

## Supplementary material

Supplemental Figure 1 Flow cytometry gating strategies for B-cell and T-cell analyses.

## Abbreviations

BCR: B-cell receptor
BLNK, B-cell linker protein (synonyms: SLP-65, BASH: BCA)
Breg: regulatory B-cell
KO: knockout
LGL: large granular lymphocyte
NK-LGL: NK-cell LGL leukemia
PRCA: pure red cell aplasia
T-LGL: T-cell LGL leukemia
Treg: regulatory T-cell
WES: whole exome sequencing.
