# Supplementary figures and images for: BLNK mutation associated with T-cell LGL leukemia and autoimmune diseases: Case report in hematology
Source: Front Med (Lausanne). 2022 Nov 16;9:997161. doi: 10.3389/fmed.2022.997161 (PMC9709112; doi:10.3389/fmed.2022.997161)

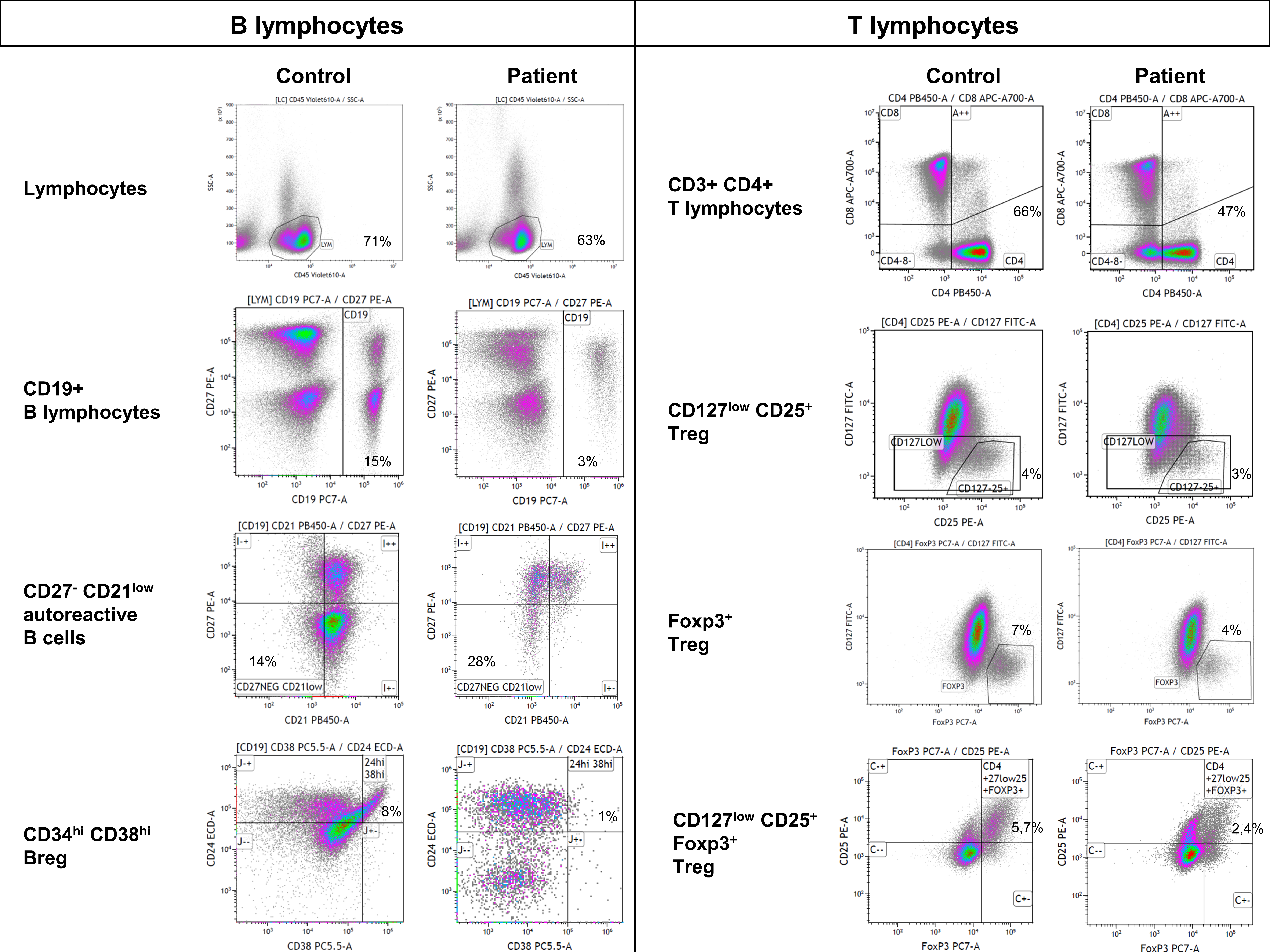

Supplement: Supplemental Figure 1 — Flow cytometry gating strategies for B-cell and T-cell analyses. [file Image_1.TIF]
